# Influence of ambient light on the accuracy of different face scanning methods: an in-vitro study

**DOI:** 10.1186/s12903-025-05594-2

**Published:** 2025-02-13

**Authors:** Paul Ulrich Keil, Florian Beuer, Alexey Unkovskiy, Ece Atay, Marie-Elise Jennes

**Affiliations:** 1 Charité – Universitätsmedizin Berlin, Department of Prosthodontics, Geriatric Dentistry and Craniomandibular Disorders, Assmanshauser Straße 4-6, Berlin, 14197 Germany; 2 Department of Dental Surgery, Sechenov First Moscow State Medical University, Bolshaya Pirogovskaya Street, 19c1, Moscow, 119146 Russia

**Keywords:** Face scanner, RMSE, Ambient light, In-vitro, Accuracy, Digital dentistry

## Abstract

**Background:**

Face scanners provide a viable method for capturing a patient’s face geometry. To optimize their accuracy, influencing factors, like the ambient light, need to be examined.

**Methods:**

A human head model with eight pins attached to its surface was used to investigate the accuracy of four face scanning methods (Face Hunter, iPad, Medit i700, single camera photogrammetry) under three illumination levels (500 lx, 5000 lx, 20 000 lx). An industrial CT scan was used as reference. Two alignment-areas – full face (AL-FF) and spheres (AL-KG) and two investigation areas – center face (UB-CF) and full face (UB-FF), were used during the examination. The root-mean-square-error (RMSE) was employed as a measure. Separated by trueness and precision, a one-way ANOVA was performed with post hoc Games-Howell tests for each scanning method.

**Results:**

All scanners showed significant differences between the illumination levels. For most test groups, the Face Hunter acquired its lowest RMSE values under 500 lx. The same can be said for the Medit i700, even though for trueness, differences to 5000 lx were random. Single camera photogrammetry performed better at higher illumination levels, but only random differences between 5000 lx and 20 000 lx were seen. For the iPad, different results for optimal illumination were found regarding trueness and precision, as well as the investigation areas. All accuracy results were labelled as highly reliable, except for the iPad´s trueness results.

**Conclusion:**

Scanner-dependent influence of ambient light was shown in this in-vitro study. Face Hunter and Medit i700 performed better under a darker illumination of 500 lx, whereas single camera photogrammetry needed brighter lighting. For the iPad no tested lighting situation showed clear advantages.

## Background

Modern dentistry is heavily impacted by an advancing trend to move workflows into a digital space. Therefore, it is necessary to virtually document patients’ individual information, as it is done with intraoral scanners [1, 2]. To capture the soft tissue of a head, face scanners are a viable method.

Most modern face scanners use one of the following three methods: laser light technology, structured light technology, and stereophotogrammetry [3, 4]. Laser light technology is made up of various scanner types based on different working principles, utilizing a laser beam. They can be divided into time-of-flight- (ToF), phase shift- and triangulation-based scanners. The most suitable scanner types for close range, and therefore face scanning, use triangulation to compute the distance between a laser-emitter and the reflecting surface [5].

Structured light scanners project a light pattern on the target’s surface, which is deformed by the 3D nature of the object. The scanner is able to capture the deformed pattern and compute a digital model by comparing it to the original [6, 7].

Similar technology is used in the TrueDepth system employed by Apple in some of their devices, where it enables facial recognition. In contrast to the mentioned structured light scanners, the Apple devices use an infrared projector in combination with infrared cameras to capture the target. With suitable applications (apps), this can also be used for scanning purposes [6, 8].

Stereophotogrammetric systems use at least two cameras to simultaneously capture the object from different perspectives [7]. A Software is used to orientate the photographs to one another by using distinctive points visible in at least two of them. In a similar method, called single camera photogrammetry, only one camera is used to capture the object from different perspectives. It utilizes a so-called structure-from-motion algorithm to reconstruct the three-dimensional camera position of each photograph [9]. Intraoral scanners have also been described in the context of digitizing parts of the face [10]. Optical scanners using visible light to capture surfaces can be influenced by ambient light, as it disturbs the pattern projected by the scanners. On the other hand, passive methods, like single camera photogrammetry, are dependent on ambient light and can profit from increased illuminance levels.

The information provided by these scanners can be used in multiple fields of dentistry, like prosthodontics, orthodontics, and maxillofacial surgery [6, 11]. They benefit the communication between both patient and dentist, as well as technician and dentist. Furthermore, the predictability of a treatment outcome can be supported by integrating facial information into simulations [8]. The data can also be used in the process of creating facial prostheses [10]. However, to accomplish all these tasks, they need to provide a certain accuracy.

Accuracy consists of trueness and precision. Trueness refers to the ability of a scanner to replicate an object as close to its original geometry as possible. Precision, on the other hand, describes the closeness between scans of a single scanner that were performed under the same conditions [12]. Previous studies have compared linear measurements obtained through direct anthropometry with corresponding measurements taken from 3D face scans. These studies found comparable accuracy between the two methods [6, 11, 13]. To examine the accuracy of face scanners, some studies have compared them with previously established face scanners, like the 3dMD-face or the Vectra H1 [8, 13, 14]. Others have employed extraoral scanners, like the D2000, the Faro Design ScanArm 2.0, or the Classic iCAT, to create reference scans [10, 15, 16]. Some studies have also employed original model files as a reference, used to 3D-print a head model for testing [6, 7].

The accuracy of scanners can be influenced by different factors, whose exact impact needs to be known for an optimal application. One of these factors is the ambient light [17]. In dental practice, a minimal illumination of 500 lx should be provided. In other areas closer to the working field and in the working field itself, brighter lighting of 5000 lx up to 15 000 lx is required [18, 19]. Windows can also influence the ambient light, direct sunlight reaching illumination levels up to 100 000 lx.

The impact of ambient light on the accuracy of intraoral scanners has been part of different investigations [17, 20–23]. The same should be done with face scanners, as the face is even more exposed to different illuminances. Thus, information could be provided regarding optimal illumination for a scanner, which could improve its accuracy by creating an optimized environment. To the authors’ knowledge there has only been one study employing a face scanner in a similar setting [24]. Hence the aim of this in-vitro study was to examine the impact of ambient light on the accuracy of different face scanning methods. The null hypothesis postulated that scanner accuracy would not be significantly influenced by variations in ambient light conditions.

## Materials and methods

### Setup

**Fig. 1:**
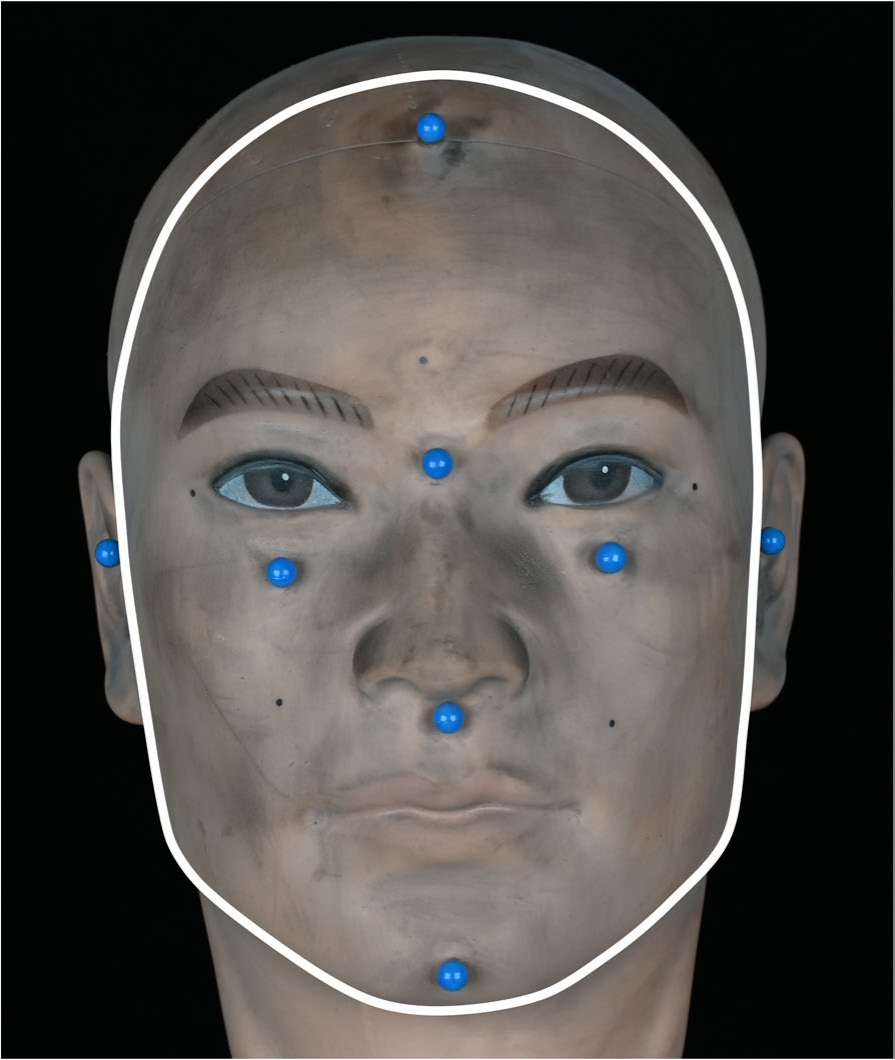
Dummy head with eight pins attached to its surface, which together form the sphere alignment area. The white outline indicates the border of the full face alignment area

A human head model, created for cosmetology, was used as a test subject. Eight pins (Stylex Schreibwaren GmbH, Bad Bentheim, Lower Saxony, Germany) were attached to its surface to provide additional geometries for alignment (Fig. 1).

**Fig. 2:**
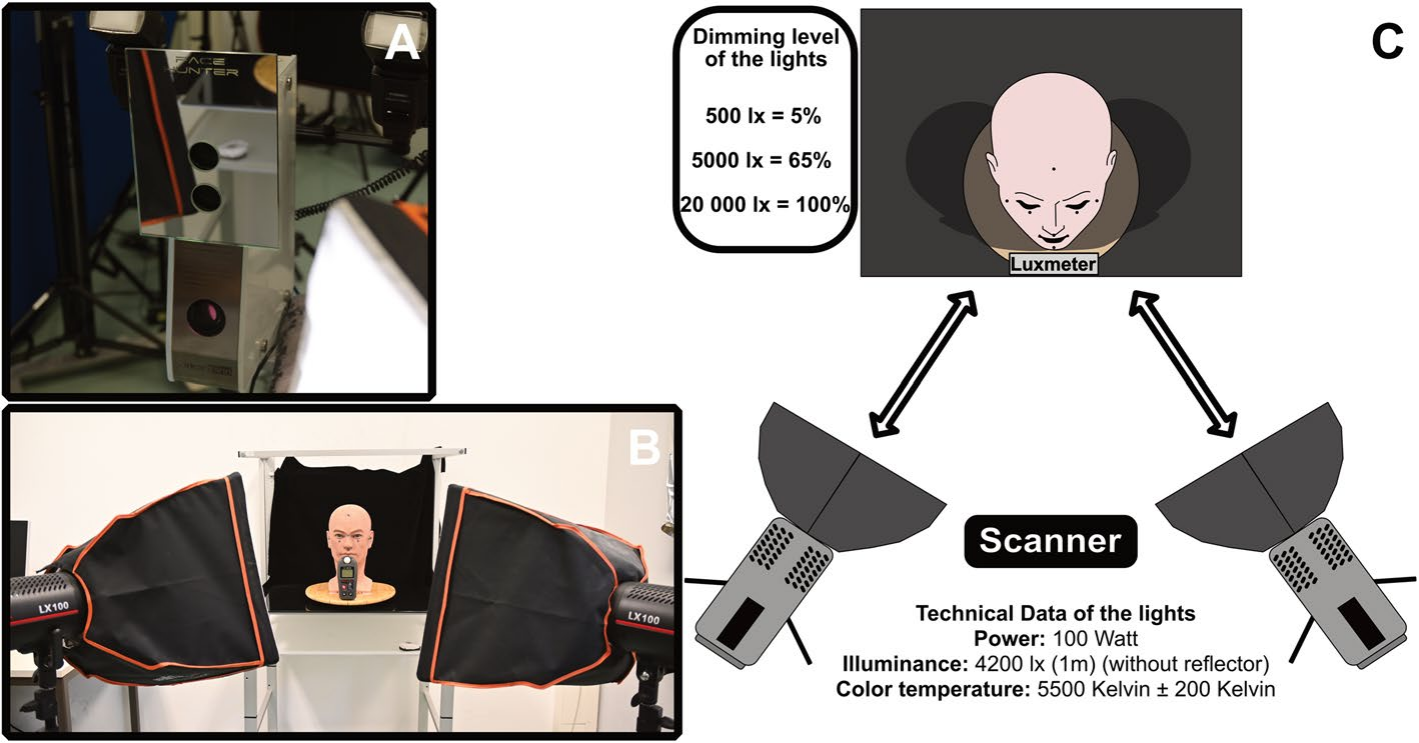
**A** Face Hunter scanner; **B** study setup; **C** schematic illustration of the study

To provide the dummy head’s surface with more details, like skin pores, scars or other variations, a dark color spray (Batiste Dunkel, Church & Dwight Deutschland GmbH, Frankfurt am Main, Hesse, Germany) was carefully applied [25–27]. To create a reference model, an industrial grade computed tomography (CT) scan, using the TomoScope HV Compact 300 kV (Werth Messtechnik GmbH, Gießen, Hesse, Germany), was performed by the company HEMA-CT (HEMA-CT Q-Technologie und Messtechnik GmbH, Uhingen, Baden-Württemberg, Germany). Two light-emitting diode (LED) lights, the LX100 (Shanghai Jinbei Photographic Equipment Industry Co., Ltd., Shanghai, China) with a color temperature of 5500 Kelvin (K) were used to achieve the three illuminance levels, which were investigated in this study: 500 lx, 5000 lx and 20 000 lx. As these lights use fluorescent white LEDs, it is unlikely that they emit any infrared light. To set up the different ambient light situations, the LEDs were dimmed and their distance to the head model changed. During the adjustment of the illumination, a digital luxmeter (Digital 20000Fc, Neoteck, Hong Kong, China) was placed directly in front of the head model to monitor the ambient light (Fig. 2).

### Data acquisition

Four face scanning methods were employed. The Face Hunter (Zirkonzahn GmbH, Gais, South Tyrol, Italy) is based on structural light technology and projects a stripe pattern of visible light onto the target. Each scan consisted of three shots taken at different angles. First, a front-facing capture was created, followed by two shots where the head was rotated left and right by 45°. The second scanner used was an iPad Pro, 11″, 3rd-generation (Apple Inc., Cupertino, California, USA; iPadOS 16.6), in combination with the Heges 3D app (Marek Simonik, Version 1.7). The app allows the user to create 3D scans using either LiDAR technology, which is based on the ToF principle, or the TrueDepth camera. In this study, the latter was employed for scanning purposes, with the following settings: data were exported using centimeters as the unit of measurement; color improvement was set to highest; the new scanning method was activated; precision was set to 0.5 mm; and range was set to the highest possible value. During the scan, the head remained in a front-facing position while the device was manually maneuvered around it, from ear to ear and from chin to forehead. The next scanner was the Medit i700 (Medit, Seoul, South-Korea; Software: Medit Link 3.1.4), which is an intraoral scanner using a visible structured light pattern, in this case a dot pattern, to capture the target’s surface. The scan path started in the mouth and chin region, after which the scanner was moved upward to capture the nose, eyes, and parts of the cheeks. For the single camera photogrammetry, a Nikon Z6 (Nikon Corporation, Tokyo, Japan) was used, in combination with a Nikkor lens (AF-S, Nikkor, 35 mm, 1:1.8). The following camera settings were employed: ISO-value = 100, aperture = f8, shutter speed = 1/4 (500 lx), 1/40 (5000 lx), 1/100 (20 000 lx). A total of 40 photographs, at five horizontal positions around the head, were taken for each scan, which consisted of five overview-shots with the complete head in frame, followed by 35 close up shots, with seven shots at different heights per horizontal position. The photographs were processed with the photogrammetry software Agisoft Metashape (Agisoft LLC, St. Petersburg, Russia; Version 1.7.5), running on a MacBook Pro M1, 13″ (Apple Inc., Cupertino, California, USA; macOS Ventura 13.5).

### Data analysis

For evaluation purposes, Geomagic Control X (3D Systems, Rock Hill, South Carolina, USA; Version 2023.0.0) was employed, running on a desktop computer (Windows 10 Pro, Version 22H2). The root mean square error (RMSE) was used. Trueness was investigated by comparing the test scans with the reference scan. For every scanning method and illumination level, the scan with the lowest RMSE value was selected as the reference in the following precision analysis, during which it was compared to the remaining scans in their individual scanning method and illumination level groups.

**Fig. 3:**
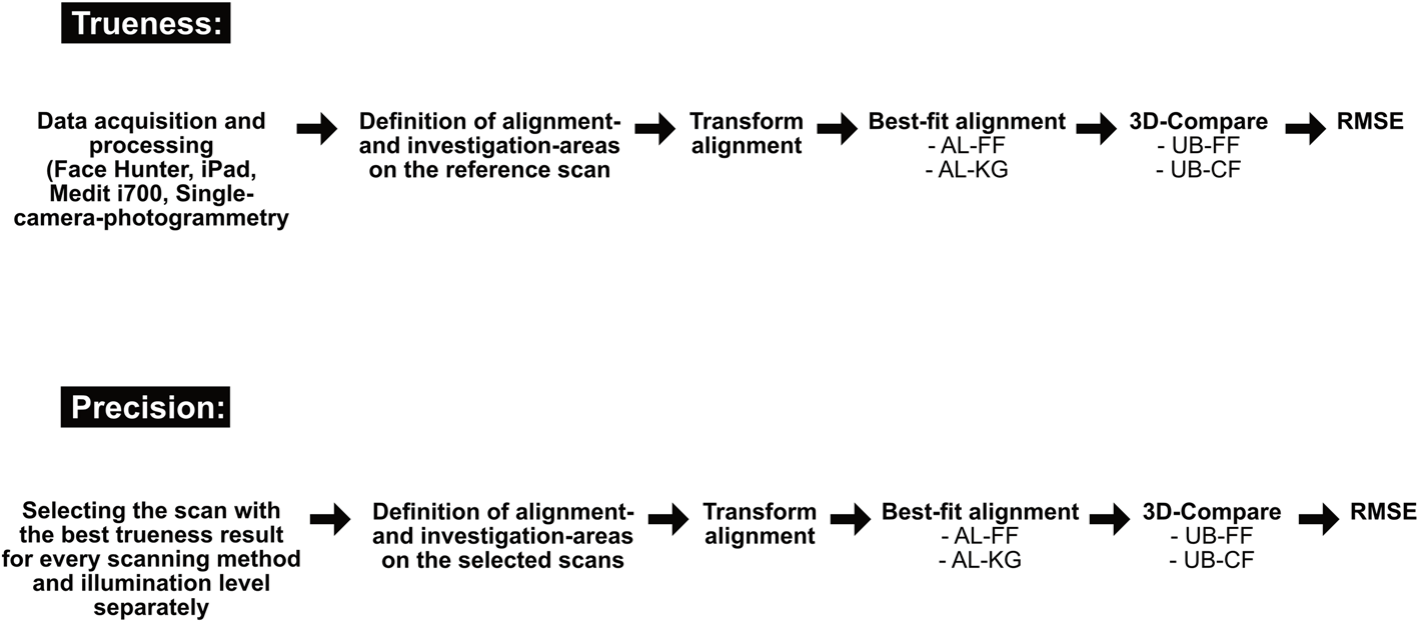
Data analysis workflow diagram

The transform alignment function was employed for an initial rough alignment, after which the best-fit algorithm was performed using two different alignment-areas. The first area covered the whole face, with exception of the pins attached to the head’s surface (AL-FF). These pins and their surrounding areas formed the second alignment area (AL-KG). Another distinction was made between two investigation areas, where one covered the whole face (UB-FF) and the other focused on the center part of it (UB-CF). The 3D-compare function was employed to analyze the scans and the creation of color maps (Fig. 3).

### Statistical analysis

To estimate the number of scans necessary for this study, five pilot scans per method were performed. Using a power of 80 percent and an alpha of 0.05 for the analysis, different numbers of scan repetitions were selected for each method: Face Hunter (*n* = 26), iPad (*n* = 45), Medit i700 (*n* = 25), single camera photogrammetry (*n* = 50). All acquired data were statistically analyzed by using SPSS statistics (SPSS inc., Chicago, Illinois, USA; Version 29.0), running on a desktop computer (Windows 10 Pro, Version 22H2). Evaluation of the alignment areas and a comparison between the different scanning methods were done visually by using error bars with 95 percent confidence intervals of the mean values. If the bars did not overlap, a significant difference could be assumed. Otherwise, the differences were only random. To evaluate the illumination levels and investigation areas, one-way ANOVA with post hoc Games-Howell tests were conducted separately for every scanning method. These investigations were also performed separately for trueness and precision. The significance threshold was defined as *p* < 0.05.

## Results

### Alignment-area investigation

**Fig. 4:**
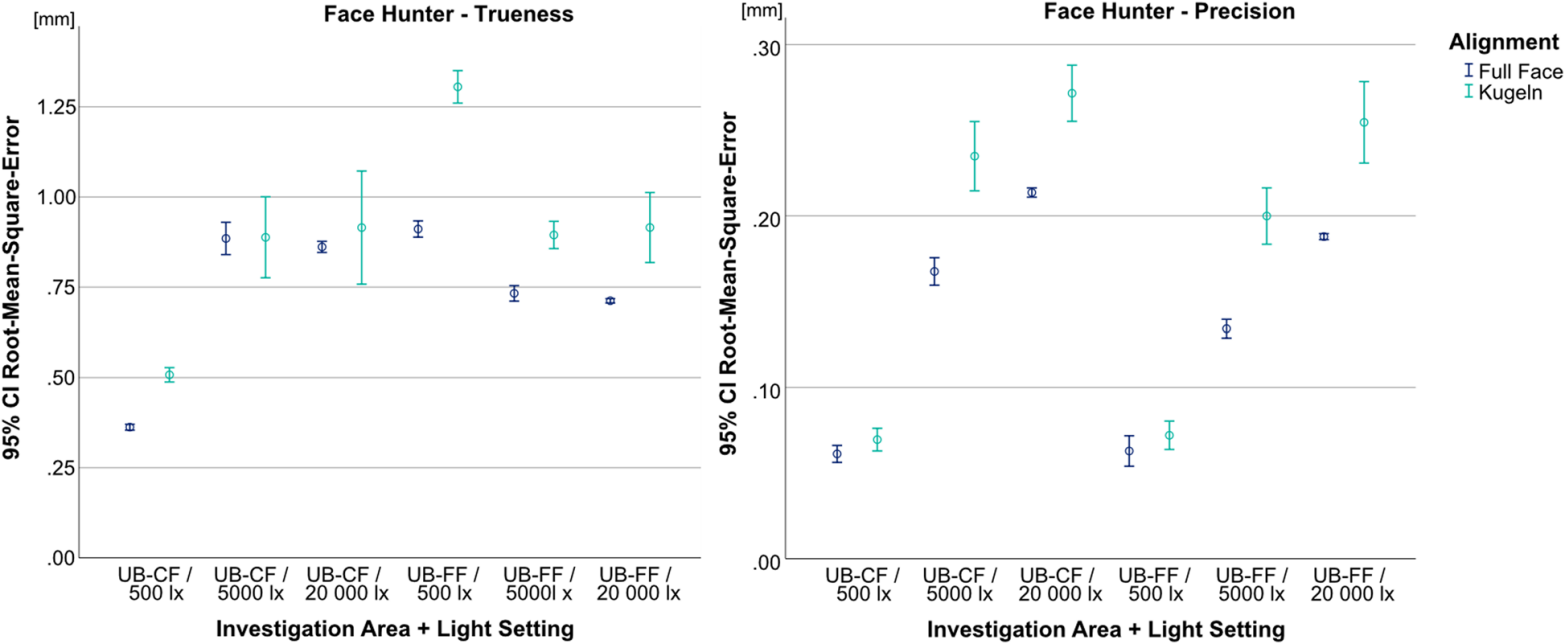
Comparison of alignment areas split by trueness (left) and precision (right) for Face Hunter; y-axis = RMSE (in mm), x-axis = illumination levels + investigation areas, circles = mean value, bars = 95% confidence interval of the mean value

All scanners had difficulty capturing the spheres, which led to deformations or, in the case of the Medit i700, to an incomplete depiction of these areas. Comparisons of the alignment-areas mostly resulted in significantly better results for the AL-FF or only produced random differences between the two areas. As an example, the comparison of alignment areas for the Face Hunter is displayed in Fig. 4. Based on these results, only AL-FF was considered during further investigations.

### Quantitative analysis

Concerning the ambient light, different results were found for the investigated methods. An overview of the RMSE values of the different scanners is given in Tables 1 and 2.

**Table 1:**
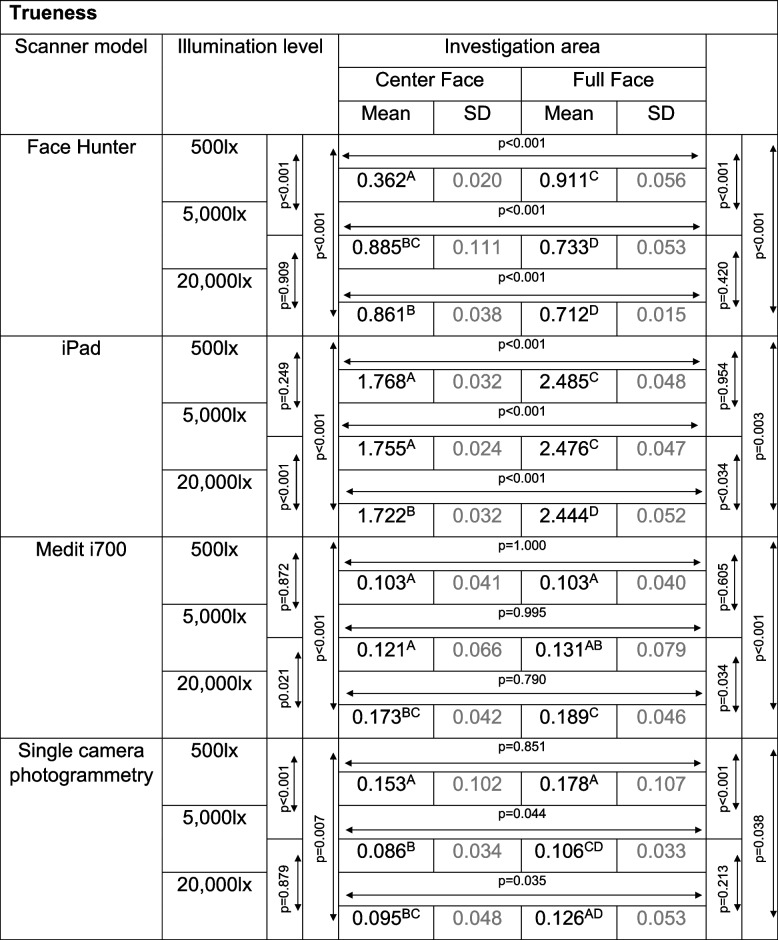
Overview of RMSE values in [mm] for trueness results

Separated by scanner model, illumination level and investigation area; between values with the same superscript letters, only random differences were found; if no letters are the same, the values’ differences are significant (*p*<0.001) (scanner models need to be viewed independently); *SD* standard deviation

**Table 2:**
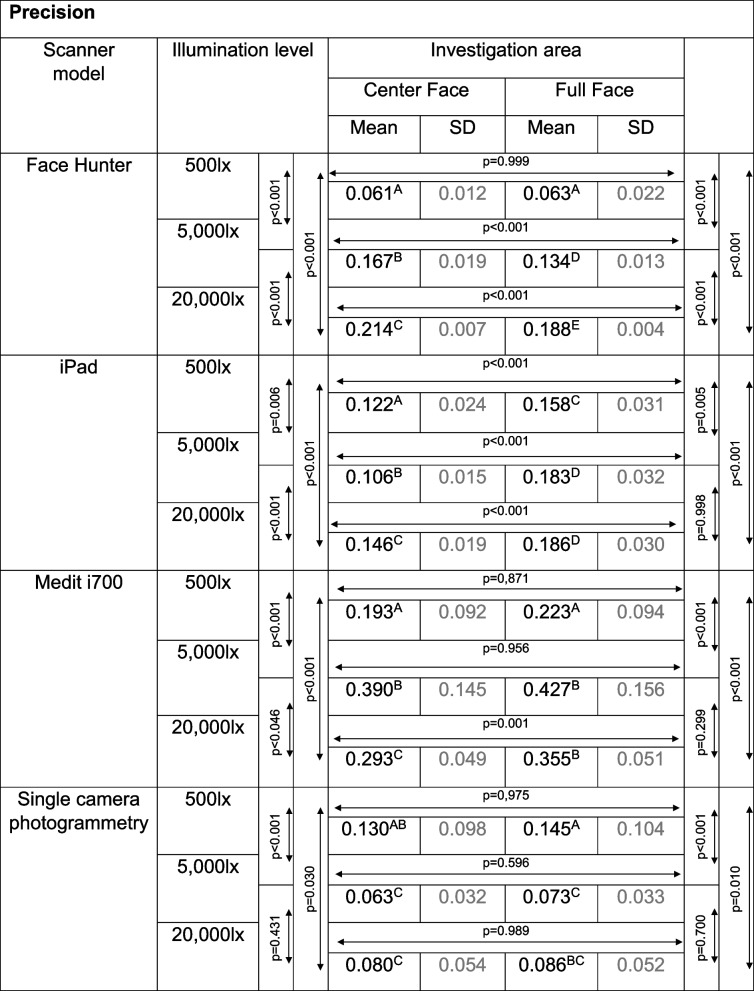
Overview of RMSE values in [mm] for precision results

Separated by scanner model, illumination level and investigation area; between values with the same superscription letters, only random differences were found; if no letters are the same, the values’ differences are significant (*p*<0.001) (scanner models need to be viewed independently); *SD* standard deviation

**Fig. 5:**
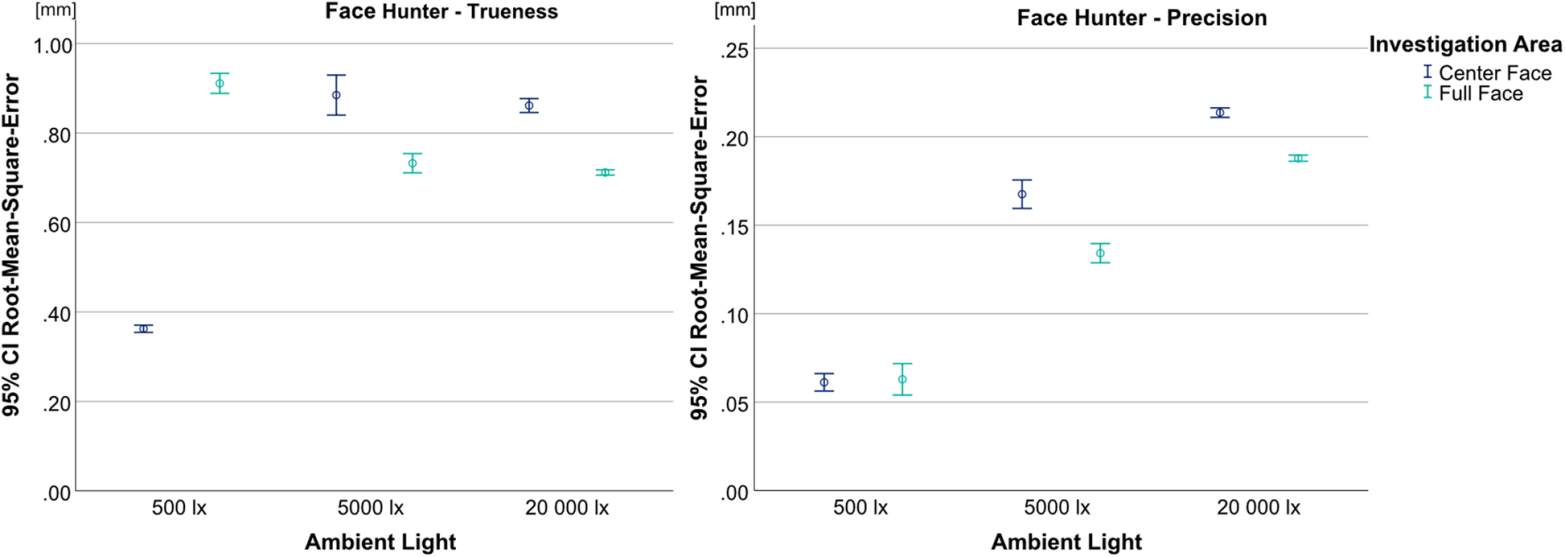
Comparison of illumination levels and investigations areas split by trueness (left) and precision (right) for Face Hunter; y-axis = RMSE (in mm), x-axis = illumination levels, circles = mean value, bars = 95% confidence interval of the mean value

For the Face Hunter, the ANOVA yielded statistically significant results, for both trueness (*p* < 0.001, F = 319.803) and precision (*p* < 0.001, F = 507.876) (Fig. 5). Regarding the trueness data, the scanner showed statistically significant lower values for the UB-CF under an illumination of 500 lx (0.362 mm) compared to the other lighting situations (5000 lx = 0.885 mm; 20 000 lx = 0.861 mm) (*p* < 0.001). In contrast, for the UB-FF, 500 lx (0.911 mm) led to statistically significant higher RMSE values compared to the other illumination levels (*p* < 0.001), with only random differences between 5000 lx (0.733 mm) and 20 000 lx (0.712 mm) (*p* = 0.420). With regard to precision, both investigation areas showed significantly lower RMSE values at 500 lx (UB-CF = 0.061 mm; UB-FF = 0.063 mm) (*p* < 0.001).

**Fig. 6:**
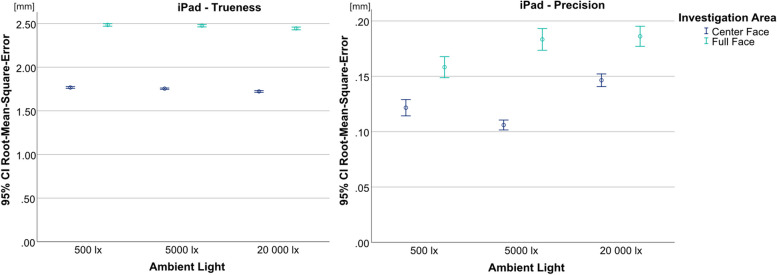
Comparison of illumination levels and investigations areas split by trueness (left) and precision (right) for Medit i700; y-axis = RMSE (in mm), x-axis = illumination levels, circles = mean value, bars = 95% confidence interval of the mean value

With the iPad, differing conclusions could be drawn for trueness (*p* < 0.001, F = 4289.084) and precision (*p* < 0.001, F = 67.997); nevertheless, significant results between lighting situations and investigation areas were found for both (Fig. 6). Regarding trueness, the lowest values were achieved with 20 000 lx (UB-CF = 1.722 mm; UB-FF = 2.444 mm). Concerning the precision results, the lowest values with UB-CF were achieved with 5000 lx (0.106 mm), whereas with UB-FF 500 lx (0.158 mm) showed the lowest results. For all test groups, UB-CF produced significantly lower RMSE values then UB-FF.

**Fig. 7:**
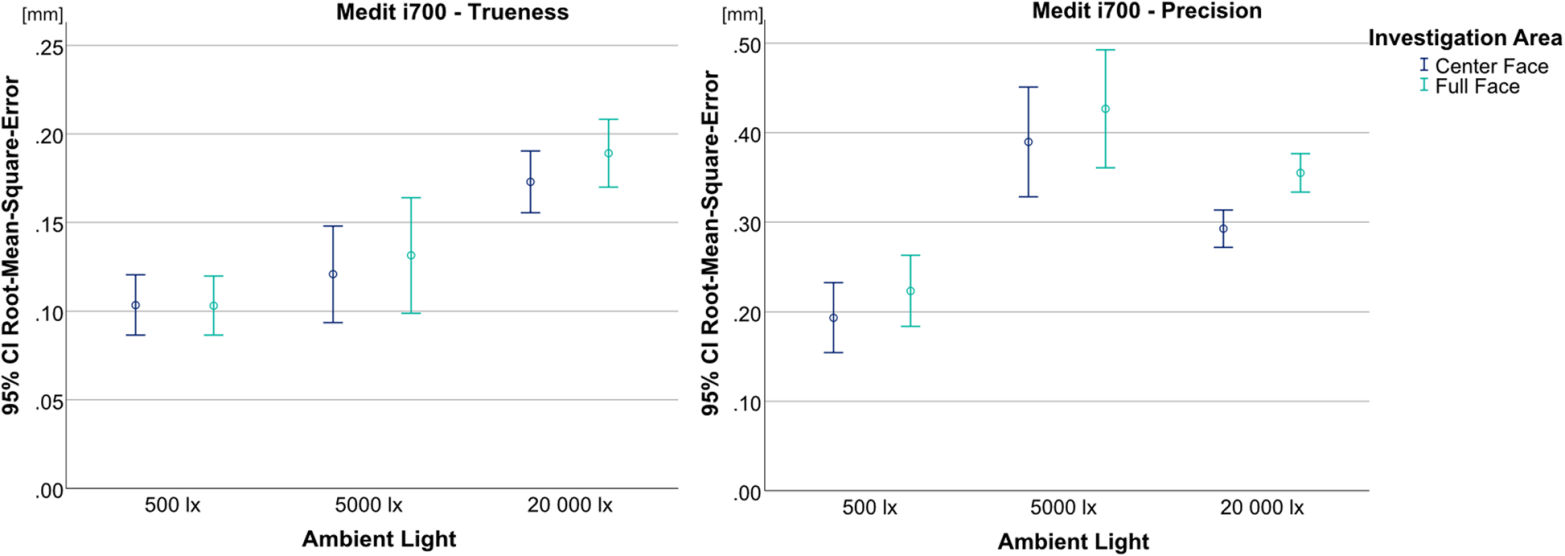
Comparison of illumination levels and investigations areas split by trueness (left) and precision (right) for Face Hunter; y-axis = RMSE (in mm), x-axis = illumination levels, circles = mean value, bars = 95% confidence interval of the mean value

The ANOVA for the Medit i700 showed statistically significant differences for both trueness (*p* < 0.001, F = 11.085) and precision (*p* < 0.001, F = 18.389) (Fig. 7). Like the Face Hunter, it achieved its lowest RMSE values for both trueness and precision at 500 lx. In terms of trueness, only random differences were found between 500 lx (UB-CF = 0.103 mm; UB-FF = 0.103 mm) and 5000 lx (UB-CF = 0.121 mm; UB-FF = 0.131 mm) for the UB-CF (*p* = 0.872) and UB-FF (*p* = 0.605). As regards precision, 500 lx (UB-CF = 0.193 mm; UB-FF = 0.223 mm) showed significantly lower results in comparison to the other two illumination levels.

**Fig. 8:**
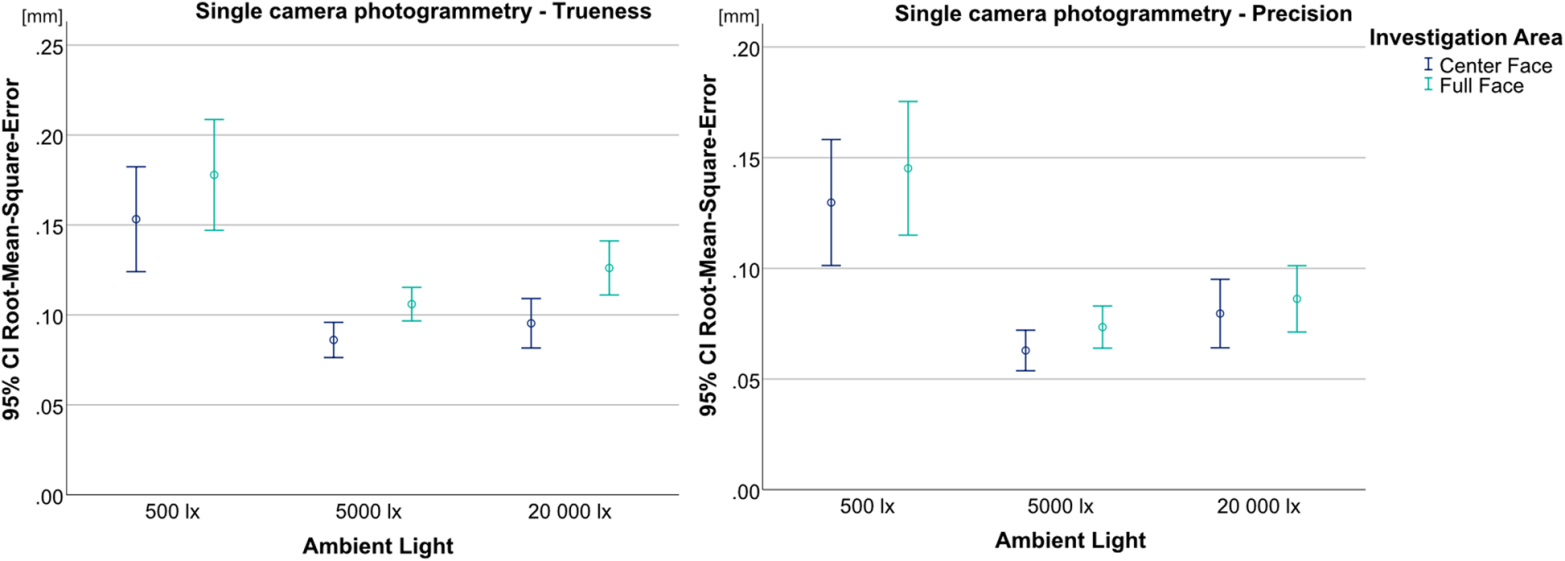
Comparison of illumination levels and investigations areas split by trueness (left) and precision (right) for single camera photogrammetry; y-axis = RMSE (in mm), x-axis = illumination levels, circles = mean value, bars = 95% confidence interval of the mean value

Regarding the single camera photogrammetry, the ANOVA showed statistically significant results for trueness (*p* < 0.001, F = 12.976) and precision (*p* < 0.001, F = 11.481). 500 lx (trueness: UB-CF = 0.153 mm; UB-FF = 0.178 mm; precision: UB-CF = 0.130 mm; UB-FF = 0.145 mm) produced significantly higher values than the other two illumination levels (Fig. 8). Between 5000 lx (Trueness: UB-CF = 0.086 mm; UB-FF = 0.106 mm; Precision: UB-CF = 0.063 mm, UB-FF = 0.073 mm) and 20 000 lx (trueness: UB-CF = 0.095 mm, UB-FF = 0.126 mm; precision: UB-CF = 0.080 mm; UB-FF = 0.086 mm), only random differences could be found, as well as between the two investigation areas for every illumination level.

Comparing only the best results of each method, the overall lowest RMSE values for trueness were achieved with the Medit i700 (UB-CF = 0.103 mm, UB-FF = 0.103 mm) and the single camera photogrammetry (UB-CF = 0.086 mm, UB-FF = 0.106 mm), with only random differences between the two. The iPad produced the highest values (UB-CF = 1.722 mm, UB-FF = 2.444 mm), while the Face Hunter (UB-CF = 0.362 mm, UB-FF = 0.712 mm) falls between these two groups.

Regarding precision, the lowest RMSE-values were achieved with the Face Hunter (UB-CF = 0.061 mm, UB-FF = 0.063 mm) and, again the single camera photogrammetry (UB-CF = 0.063 mm, UB-FF = 0.073 mm). Only random differences can be seen between the methods. The highest values were produced by the Medit i700 (UB-CF = 0.193 mm, UB-FF = 0.223 mm). The iPad’s results (UB-CF = 0.106 mm, UB-FF = 0.158 mm) fall between these two groups.

### Qualitative analysis

**Fig. 9:**
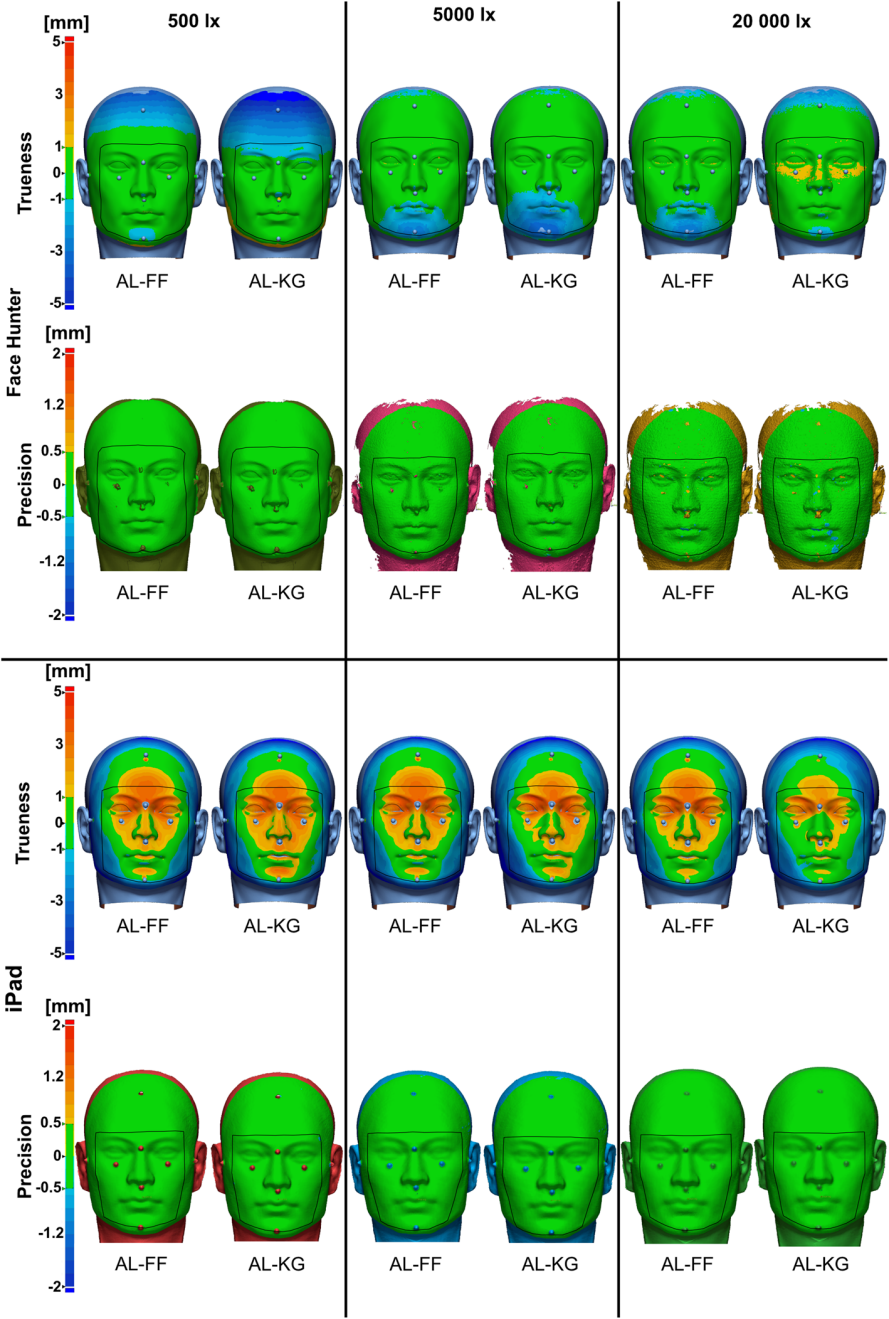
Color maps for Face Hunter and iPad; trueness tolerance level = ± 1 mm, precision tolerance level = ± 0.5 mm; yellow to red color indicates positive deviations; light-blue to dark-blue indicates negative deviations; green indicates areas that are in tolerance; the border of UB-CF is presented by the black outline

**Fig. 10:**
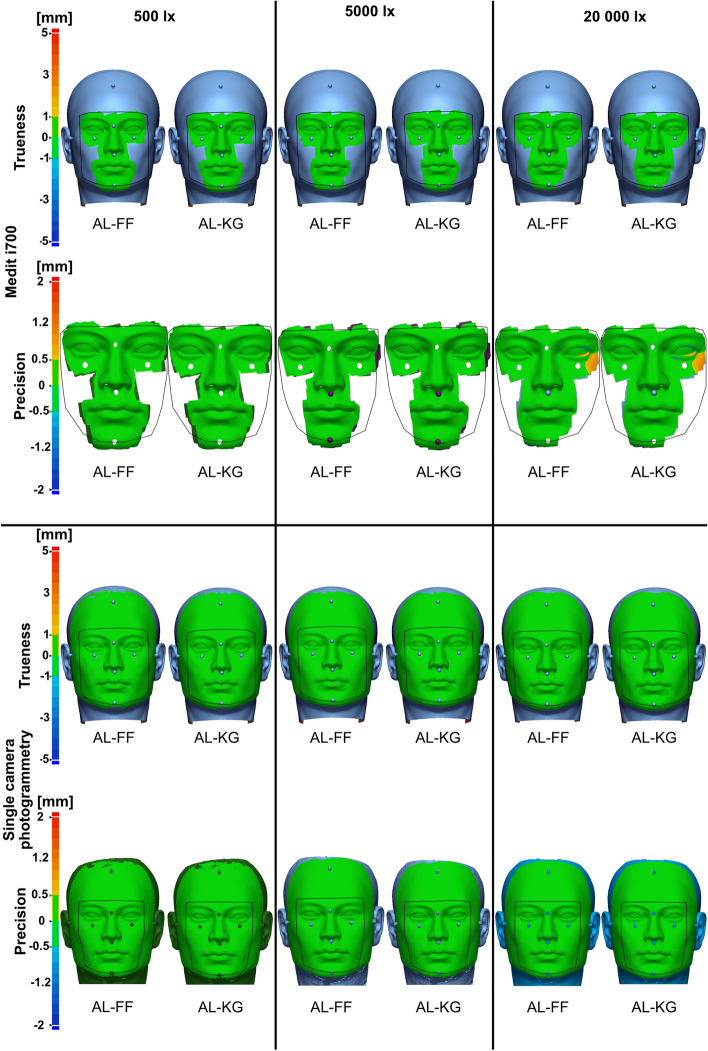
Color maps for Medit i700 and single camera photogrammetry; trueness tolerance level = ±1 mm, precision tolerance level = ± 0.5 mm; yellow to red color indicates positive deviations; light-blue to dark-blue indicates negative deviations; green indicates areas that are in tolerance; the border of UB-CF is presented by the black outline

Color maps for trueness and precision results are displayed in Figs. 9 and 10. At 500 lx, the Face Hunter showed negative deviations in the forehead area, whereas at brighter lighting levels, these were considerably smaller. At 5000 lx and 20 000 lx, negative deviations can be found in parts of the chin. With respect to precision, no deviations were visible, but a difference in mesh quality was recognized. At 500 lx the model appeared smooth, whereas brighter illuminations showed a rougher surface and incomplete depiction of the outer edges of the face, including ears, forehead, etc. Regarding trueness, the iPad scans exhibited positive deviations in the central facial region, which turned increasingly negative toward the peripheral areas of the scan, with a round area between the two, which was within the acceptable tolerance range. These deviations were present at all illumination levels. The Medit i700 and single camera photogrammetry showed no visible deviations in the color maps.

## Discussion

Face scanners display a promising technique to capture the soft tissue profile of a human face without radiation exposure. To integrate this information into a functioning workflow, a certain degree of accuracy needs to be provided by the scanners. Otherwise, errors can be introduced into the different application areas, like treatment simulation with digital smile design or the manufacturing of facial protheses.

The accuracy of a scanner can be influenced by environmental factors, such as ambient light or scanning strategy. To achieve optimal performance of a device, these factors need to be part of research. Regarding ambient light, this study found a scanner-dependent influence of different illumination levels. Therefore, the null hypothesis was rejected.

The differing effects of ambient light on the various scanning methods used were also concluded in other studies that investigated intraoral scanners [20, 21]. Difficulties arise in the interpretation of the accuracy results, because different thresholds are regarded as clinically significant. Some authors describe errors up to 3 mm as clinically acceptable [28], while others already draw the border at 1.5 mm [29]. Aung et al. [30] proposed different categories of deviation, where errors up to 1 mm were described as highly reliable, between 1 and 1.5 mm as reliable, between 1.5 and 2 mm as moderately reliable and above 2 mm as unreliable. This definition was also adopted in other studies [8, 13], which therefore described deviations above 2 mm as unreliable.

In the present study, the Face Hunter achieved accuracy results, that can be categorized as highly reliable. Another study that tested the Face Hunter’s accuracy by comparing it to a line laser scanner showed trueness values of 0.117 mm over the whole face [15]. These results are not in accordance with the present study and could be explained by differences in the study setup or scanning procedure. Differences in the dummy heads used could also account for the varying results.

Regarding ambient light, the Face Hunter achieved its best results at 500 lx, except for the UB-FF’s trueness results, where it produced the highest RMSE-values. This could be explained by the negative deviations and artifacts visible in the color-maps. At 500 lx, deviations mostly occurred in the forehead area and therefore mainly influenced the UB-FF. This could be due to insufficient illumination, because at brighter levels, deviations in this area were much smaller.

Similar results were found by Thongma-Eng et al. [24], which to the authors’ knowledge is the only study available to investigate the effect of ambient light on the accuracy of a face scanner. Amongst other methodological differences, the illumination levels in their study were not measured in lux, which limits comparability. Only the natural light group was stated with a value of 500 lx. The examined scanner, an Einscan Pro 2X Plus, performed best at this illumination level. This is comparable to the Face Hunter’s results, as both scanners utilize visible structured light to capture the target’s surface. Hence, higher illumination levels can disturb the projected light pattern as well as the scanner’s sensors.

In the present study the iPad’s trueness results ranged from 1.722 mm to 1.768 mm for the UB-CF and from 2.444 mm to 2.485 mm for the UB-FF. Deviations above 2 mm can highly influence treatment results that build upon the face scanner’s information – e.g. 3D smile design, where it could lead to noticeable discrepancies [31].

These results are not in accordance with other studies investigating the accuracy of smart devices in combination with scanning apps. Among other extraoral scanners, Loy et al. [6] investigated different kinds of scanning applications and showed trueness results below 0.5 mm for all of them. At 0.28 mm, even lower trueness results were found by Rudy et al. [14] in an in-vitro investigation. These differences could be due to the reference model used, because the two studies employed different methods to create them. Loy et al. [6] printed a 3D head-model and utilized the digital model as the reference, while Rudy et al. [14] used another face scanner. This is supported by the fact that the precision results of both studies are in accordance with the present study. Another reason for the discrepancies in trueness results could be the application of different iPad model generations or Apple devices, like the iPhone. Information on how these hardware variations could influence trueness results, is not available and could be part of future investigations.

The ambient light investigation showed that different illumination levels produced the best performance for trueness and precision. For trueness, the lowest results were achieved at 20 000 lx. Regarding precision, 5000 lx led to the lowest results for the UB-CF, and 500 lx for the UB-FF. Based on this, no ambient light setting can be deemed superior for the iPad.

This is in accordance with the fact that the TrueDepth camera uses infrared lighting to capture the surface. Therefore, it should not be influenced by the employed LED lights. The variations between the ambient light groups could be the result of changes in the natural sunlight or other sources of infrared light that were not accounted for. To the authors’ best knowledge, comparable results are not available, as this has never been investigated before.

The accuracy of the Medit i700 can be categorized as highly reliable. To the authors’ knowledge, it has not previously been tested for its accuracy when digitizing parts of a face. A comparison could be made to a study by Unkovskiy et al. [10], who, among other scanners, utilized intraoral scanners to capture models of various face parts. When compared to the values of the Medit i700 in the present study, their investigation showed similar results for the Trios 4 scanner. Furthermore, they utilized an iPhone in combination with the Heges app and, like the studies mentioned before [6, 14], found lower trueness values but similar precision results.

The Medit i700 achieved its best results at 500 lx, even though only random differences to the trueness results of 5000 lx were found. Regarding precision, 500 lx showed significantly better results than 5000 lx. Thus, like the Face Hunter, the results of the present study suggest lower illumination levels, when using the Medit i700. This is supported by the fact that both scanners project visible structured light patterns.

A study that investigated the influence of ambient light on the accuracy of the Medit i700, showed similar trueness results, as it only presented random differences between 500 and 5000 lx [22]. The authors achieved the lowest trueness values at 100 lx. Regarding precision, the lowest results were achieved at 5000 lx, which is not in accordance with our study, but could be explained by methodological differences, as they investigated implant bodies. A restriction in the scanner’s utilization was that it was not able to capture the whole face. Consequently, comparisons to other scanners are limited.

The single camera photogrammetry showed highly reliable accuracy results. Studies investigating this method in the context of face scanning are rare. Comparisons to those that do exist are difficult due to methodological differences. In a past study, the accuracy of two software providers, Agisoft Metashape and 3DF Zephyr, was investigated [16]. Deviations up to 4 mm for 3DF Zephyr and 2.3 mm for Agisoft Metashape were shown, which are a lot higher than the results presented in the present study. However, comparisons are limited because of the in-vivo setting and the employment of different equipment, different number of photographs per scan, and different camera settings.

As a passive method, the single camera photogrammetry depends on ambient light. This is supported by the study’s findings, as an illumination of 500 lx led to statistically significantly higher values compared to the other lighting levels used, between which only random differences were found. The higher RMSE values could be the result of a lower grade of detail in the photographs or the shutter speed, which was changed at different illumination levels. Brighter lighting allowed faster shutter speeds which reduced motion artifacts. Overall, the single camera photogrammetry showed promising results in terms of accuracy, that can be compared to in-vitro results of face scanners already validated [7].

Dedicated face scanners provide a viable method for capturing a patient’s facial physiognomy but are also affiliated with great expense. Therefore, their affordability for regular dental practices is limited. With the growing availability of intraoral scanners, utilization for facial scanning would provide an advantage. With the Medit i700, it was only possible to capture a part of the face, but future innovations could allow a larger scan area. With their wide availability smartphones provide an inexpensive alternative for 3D scanning. With constant improvements to smartphones and their camera systems, the scanning accuracy of such devices could also be increased. However, not all devices are authorized for medical use. Apart from the TrueDepth and LiDAR technology employed by Apple devices, smartphones can also be utilized for single camera photogrammetry. The cost of the single camera photogrammetry depends on the equipment and software used. Differences in the setup could also lead to different accuracy, which should be investigated in future studies. These studies should also focus on the clinical application of these methods.

One of the main limitations of our study is its in-vitro nature, which was chosen to investigate the isolated ambient light impact. Limitations resulting from this study setup are the lack of hair and reflective skin surface, resulting from sweat and sebum [10]. Another main limitation of this study is the use of the motionless head model, as the influence of motion on the accuracy of face scanners has been proven [7]. Future studies should incorporate human subjects to examine the impact of ambient light in connection with other influences, like involuntary motions.

The light measurement is a further limitation of our study, as the luxmeter could only record the illuminance in one location. Accordingly, a certain heterogeneity in the lighting of other areas, especially the sides of the dummy head, cannot be ruled out. Future studies could measure the illuminance over the whole investigation area and thereby address the poor uniformity of lighting. Another limitation of our study setup is that the sensor positions were not recorded in the case of the Face Hunter and single camera photogrammetry. Lastly, the ambient temperature was not measured during the scans.

## Conclusion

Within the limitations of this in-vitro study, the following conclusions can be drawn:

- The Face Hunter and Medit i700 showed their overall best performance under an illumination of 500 lx, as was expected because of their use of visible structured light, which is disturbed at higher illuminance levels.
- Single camera photogrammetry showed its lowest RMSE values at 5000 lx, though only random differences were found in comparison to 20 000 lx. As this is a passive method, it depends on ambient light and profits from the higher illuminance levels.
- Since the iPad’s TrueDepth system is based on infrared lighting, it should not be affected by the employed lights. This is in line with the study’s results, as no ambient light setting showed advantages over others.
- Regarding overall performance, the lowest trueness values were achieved with the Medit i700 and the single camera photogrammetry, with only random differences between the two methods.
- The lowest precision values were achieved with the Face Hunter and single camera photogrammetry, with only random differences between the two methods.

## Data Availability

The datasets generated and/or analyzed during the current study are available in the Mendeley data repository: https://data.mendeley.com/datasets/73fmxgbcw9/1. doi:10.17632/73fmxgbcw9.1.
